# LncRNA SNHG6 sponges miR-101 and induces tamoxifen resistance in breast cancer cells through induction of EMT

**DOI:** 10.3389/fonc.2022.1015428

**Published:** 2022-09-23

**Authors:** Mohammad Imran Khan, Aamir Ahmad

**Affiliations:** ^1^ Department of Biochemistry, Faculty of Science, King Abdulaziz University, Jeddah, Saudi Arabia; ^2^ Centre of Artificial Intelligence for Precision Medicines, King Abdulaziz University, Jeddah, Saudi Arabia; ^3^ Translational Research Institute, Academic Health System, Hamad Medical Corporation, Doha, Qatar

**Keywords:** tamoxifen resistance, SNHG6, miR-101, epithelial-to-mesenchymal transition (EMT), epigenetic

## Abstract

Acquired resistance is a major clinical challenge for tamoxifen-based therapy. In this study, we focused on lncRNA SNHG6 which plays a role in chemoresistance of cancer cells, but has never been investigated in the context of tamoxifen resistance. We found elevated levels of SNHG6 in tamoxifen-resistant estrogen receptor (ER)-positive MCF-7 cells (MCF7TR), relative to naïve MCF-7 cells, as well as in tamoxifen-resistant T47D cells (T47DTR), relative to naïve T47D cells, which correlated with induced vimentin, ZEB1/2 and decreased e-cadherin, thus implicating a role of EMT in SNHG6-mediated tamoxifen resistance. Downregulation of SNHG6, using specific siRNA, sensitized MCF7TR as well as T47DTR cells to tamoxifen along with markedly reduced proliferation, invasion and anchorage-independent clonogenicity. Further, SNHG6 was found to sponge and inhibit miR-101 as the endogenous expression levels of SNHG6 and miR-101 inversely correlated in paired parental and tamoxifen-resistant cells and, moreover, silencing of SNHG6 in tamoxifen-resistant cells resulted in de-repression of miR-101, along with reversal of EMT. SNHG6 expression also directly correlated with increased stem cells markers Sox2, Oct4 and EZH2. miR-101 levels, manipulated by transfections with pre/anti-miR-101 oligos, directly affected tamoxifen sensitivity of ER-positive cells with pre-miR-101 sensitizing MCF7TR and T47DTR cells to tamoxifen whereas anti-miR-101 inducing resistance of parental MCF-7 and T47D cells to tamoxifen. Further, miR-101 was found to attenuate SNHG6-mediated effects on tamoxifen resistance, EMT as well as stem cell markers, thereby making a case for SNHG6-miR-101 axis in tamoxifen resistance of ER-positive breast cancer cells. Thus, lncRNA SNHG6 is a novel modulator of tamoxifen resistance through its sponging of miR-101 and the resulting effects on EMT.

## Introduction

Breast cancer is a major cancer that mostly affects women with 287,850 new cases of invasive breast cancer estimated for the current year 2022 in the United States (1). Worldwide, approximately 2.3 million breast cancer cases were diagnosed in the year 2020 (2). There has been an increase in the newly diagnosed cases over past several years, primarily due to aggressive screening and vigilance (3), with the rate of increase around 0.5% since the mid-2000s (1). With these numbers, breast cancer ranks number one among all cancers that are diagnosed in women in the US, accounting for almost one-third of all cancer diagnoses in the women. Treatment of breast cancer patients is particularly challenging because of the many individual breast cancer subtypes. The estrogen receptor (ER)-positive breast cancers make up to 60% of all breast cancers (4) and are therefore a major subtype. The ER-positive breast cancers are managed through administration of ER antagonist tamoxifen, however, acquired resistance against tamoxifen remains a major clinical challenge (5).

Recent years have witnessed a growing interest in the involvement of non-coding RNAs in tamoxifen resistance of ER-positive breast cancers (6, 7). Various non-coding RNAs, ranging from microRNAs (miRNAs) to long non-coding RNAs (lncRNAs) have been reported to mechanistically and functionally affect the tamoxifen resistance. Our own earlier work demonstrated a role of miR-10b in the tamoxifen resistance of ER-positive breast cancers (8) and such role of miRNAs in tamoxifen resistance has been investigated by several other researchers as well (6, 7, 9). Further, lncRNAs are novel molecules of interest in terms of their possible role in the modulation of response to tamoxifen in ER-positive breast cancers (10). Among the many lncRNAs currently being investigated for their possible role in cancer drug resistance, lncRNA SNHG6 is a promising lncRNA that has been associated with increased risk of poor overall survival of cancer patients (11). High expression of SNHG6 has been demonstrated to correlate with tumor progression and poor prognosis in multiple human cancers (12). Even though a role of SNHG6 in cancer radio-resistance (13) as well as chemoresistance (14), particularly resistance against cisplatin (15) and paclitaxel (16) has been described, there is no report on it’s possible role in acquired tamoxifen resistance of ER-positive breast cancers, prompting us to plan this study. Using a paired ER-positive breast cancer cell line comprising of MCF-7 cells that are sensitive to tamoxifen along with their derivate cells that are resistant to tamoxifen (MCF7TR), we investigated the SNHG6-regulated mechanism of tamoxifen resistance. We further confirmed the findings using a parallel cell line pair comprising of tamoxifen-sensitive ER-positive T47D cells and their tamoxifen-resistant derivatives, the T47DTR cells.

## Materials and methods

### Cell lines and reagents

MCF-7 and T47D breast cancer cells were purchased from ATCC (USA) and cultured in DMEM and RPMI cell culture medium (ThermoFisher Scientific, USA), respectively, with 10% fetal bovine serum, 100 units/ml penicillin, and 100μg/ml streptomycin in a 5% CO_2_ atmosphere at 37°C. The tamoxifen resistant MCF-7 and T47D cell lines, MCFTR and T47DTR, respectively, were generated by culturing cells in their respective culture mediums supplemented with 5% FBS, antibiotics and 10^−6^ M 4-hydroxy tamoxifen (TAM), as described previously (8). TAM concentration was gradually increased over the course of six months until the final concentration was 10^−6^M.

### lncRNA downregulation

siRNAs (ThermoFisher Scientific, USA) designed against SNHG6 were transfected into MCF7TR cells using Lipofectamine RNAiMAX Transfection Reagent. Knockdown was evaluated by qRT-PCR. Our detailed preliminary validation revealed that the siRNAs were effective at 30nM final concentration and at 24 hours post-transfection, therefore, we used 30nM concentration of si-SNHG6 for our experiments. The siRNA used in our study was locked nucleic acid (LNA) modified siRNA (ThermoFisher Scientific, USA) and was used for increased potency and lower off-target effects.

### siRNA transfections

We used Lipofectamine RNAiMAX Transfection Reagent (ThermoFisher Scientific, USA) for the transfections of si-SNHG6 in MCF7TR cells for effective downregulation of the lncRNA. Cells were first seeded in 24-well plates in a total volume of 500μl in culture medium without antibiotics. Cells were seeded at a density so that they were 50% confluent the next day. On the day of transfections, siRNA-Lipofectamine complexes were prepared by diluting siRNA in 50μl of Opti-MEM ^®^ I Reduced Serum Medium without serum. In a separate tube 1μl of Lipofectamine RNAiMAX Transfection Reagent was diluted in 50μl of Opti-MEM ^®^ I Reduced Serum Medium. The contents of two individual tubes were then mixed and incubated for 20 minutes at room temperature. The contents were then added on to the individual wells without removing the overnight culture medium. The calculations were done to ensure 30nM concentration of siRNA in the total final volume of 600μl. Contents of the wells were mixed by gentle rocking and plates were transferred back to cell culture incubators. Effect of siRNA transfections were tested after an incubation of 24 hours.

### Cell growth inhibition studies by 3-(4,5-Dimethylthiazol-2-yl)-2,5-diphenyltetrazolium bromide (MTT) assay

Parental as well as tamoxifen-resistant cells were seeded overnight at a density of 5 x 10^3^ cells per well in 96-well culture plates. Thereafter, culture medium was aspirated and replaced with fresh complete culture medium containing DMSO (vehicle control) or different concentrations of tamoxifen, as indicated. After 48 hours, 25µl of 3-(4,5-dimethylthiazol-2-yl)-2,5-diphenyltetrazolium bromide (MTT) solution (5mg/ml in phosphate-buffered saline, PBS) was added to individual assay wells and incubated further for 2 h at 37°C. Upon termination, the supernatant was removed and the MTT formazan, formed by metabolically viable cells, was dissolved in DMSO (100µl) by mixing for 30 min on a gyratory shaker. The absorbance was measured at 595 nm on Ultra Multifunctional Microplate Reader (TECAN, USA).

### Apoptosis assay (Histone/DNA ELISA)

We used Cell Death Detection ELISA Kit (Roche) to detect apoptosis, as described earlier (8). Briefly, after the cells were appropriately treated, as indicated for individual experiments, the cytoplasmic histone/DNA fragments were extracted and incubated in microtiter plate modules coated with anti-histone antibody. Thereafter, peroxidase-conjugated anti-DNA antibody was used to detect immobilized histone/DNA fragments, followed by color development with ABTS substrate for peroxidase. The spectrophotometric absorbance of the samples was determined using Ultra Multifunctional Microplate Reader (TECAN, USA) at 405 nm.

### Cell invasion assay

Cell invasion was assessed using 24 well transwell permeable supports with 8 µM pores (Corning, USA). After appropriate experimental set-up, as indicated for individual experiments, cells were trypsinized and re-suspended in serum free medium before seeding into the transwell inserts coated with growth factor reduced Matrigel (BD Biosciences, USA). Bottom wells were filled with complete media. After 24 hours, cells were stained with 4 µg/ml calcein AM (ThermoFisher Scientific, USA) in PBS at 37°C for 1 h. Cells were detached from inserts by trypsinization and fluorescence of the invaded cells was read using ULTRA Multifunctional Microplate Reader (TECAN, USA).

### Anchorage-independent clonogenicity assay

Tamoxifen-resistant cells were first transfected with appropriate siRNAs (non-specific control siNS or siSNHG6), allowed to grow for 24 h and then collected by trypsinization. 3 × 10^4^ cells were then seeded in 0.5 ml of complete culture medium containing 0.3% (w/v) top agar layered over a basal layer of 0.7% (w/v) agar (with culture medium and the supplements) in 24-well plates. After 21 days of culture, colonies were counted using a phase contrast microscope (Nikon, USA).

### Prediction of miRNA targets of lncRNA SNHG6

miRNA targets of lncRNA SNHG6 were predicted using DIANA-LncBase v3 (https://diana.e-ce.uth.gr/lncbasev3), as described recently by others (17). DIANA-LncBase v3 is a reference repository that lists experimentally supported miRNA targets on non-coding transcripts. As of the date of access of DIANA-LncBase v3, the database consisted of approximately ~500,000 entries, corresponding to ~240,000 unique tissue and cell-type specific miRNA-lncRNA interactions. As per the information on database webpage, the incorporated interactions between lncRNAs and miRNAs are defined by 15 distinct low-/high-throughput methodologies, corresponding to 243 distinct cell types/tissues and 162 experimental conditions. We listed the miRNA targets of lncRNA SNHG6 without any bias for cell type or other parameters that could have affected the listing of miRNA targets in any way.

### miRNA transfections

Transfections of pre/anti-miR-101 were done using methodology previously described (8). Briefly, cells were seeded (2.5 x 10^5^ cells per well) in six well plates and transfected with pre/anti-miR-101 or non-specific pre/anti-miRNA controls (ThermoFisher Scientific, USA) at a final concentration of 200 nM, using DharmaFECT transfection reagent (Dharmacon, USA). After 48 hours of transfection, cells were passaged and transfected twice again, using the same methodology, before being used in the individual experiments.

### qRT-PCR and miRNA detection

Real-Time quantitative (q)RT-PCR analyses were also done as described previously (8). Total RNA was isolated from the cells, after the completion of individual experiments, using the mirVana miRNA isolation kit (ThermoFisher Scientific, USA). RNAse-free water was used throughout the analysis. RT^2^ First Strand Kit (Qiagen, USA) was used to synthesize cDNA first strand using 1μg RNA, to which 2μl of genomic DNA elimination mix was added, mixed and incubated 10 minutes at 42^0^C, followed by immediate transfer to ice for 1 minute. Reverse transcription mix, consisting of 5x buffer and Reverse Transcriptase, was then prepared and added to RNA which was then subjected to incubation for 15 minutes at 42^0^C. Finally, the reaction was stopped by incubation at 95^0^C for 5 minutes. The levels of miR-101 were determined using miRNA-specific Taqman probes from the Taqman MicroRNA Assay (ThermoFisher Scientific, USA). The relative amounts of miRNA were normalized to RNU6B.

### Statistical analysis

All experiments were performed a minimum of three times with triplicate repeats in individual experimental setup. To evaluate if 2 datasets were significantly different, a p value was calculated using Student *t* test or one way ANOVA assuming equal variables and 2-tailed distribution. Prior to the statistical tests, datasets were log-transformed to ensure normal distribution. In all of our experiments, the p values <0.05 were considered to be statistically significant.

## Results

### SNHG6 is elevated in tamoxifen resistant cells and positively regulates acquired resistance against tamoxifen

**Figure 1:**
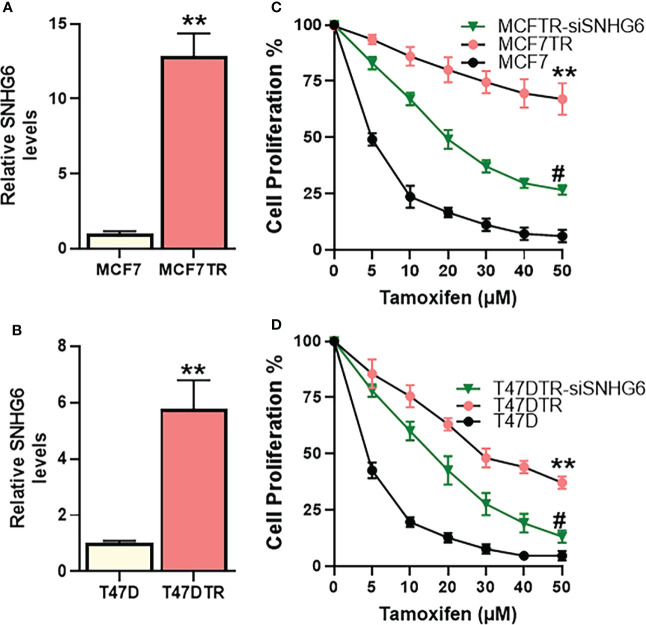
SNHG6 expression and function in tamoxifen resistant ER-positive Breast cancer cells. Expression of SNHG6 was quantitated in tamoxifen-resistant **(A)** MCF-7 cells (MCF7TR) and **(B)** T47D cells (T47DTR) by qRT-PCR. The levels of SNHG6 in parental cells (MCF-7/T47D) were assigned a value of 1 and the relative levels in tamoxifen-resistant derivatives are plotted. Cell proliferation was measured by MTT assay in paired cell lines **(C)** MCF-7/MCF7TR and **(D)** T47D/T47DTR after the cells were exposed to indicated concentrations of tamoxifen for 48 hours. Further, tamoxifen resistant MCF7TR and T47DTR were subjected to SNHG6 silencing and then treated with increasing concentrations of tamoxifen for 48 hours before the proliferation was assessed using MTT assay. **p<0.01, compared to native controls and #p<0.01, compared to respective resistant cells without SNHG6 silencing.

We started our investigation by assessing the relative levels of lncRNA SNHG6 in parental MCF-7 cells and their tamoxifen resistant counterparts, the derived MCF7TR cells. SNHG6 levels were more than 12-folds elevated in the MCF7TR cells, compared to MCF-7 cells (**Figure 1A**) indicating a correlation of lncRNA SNHG6 with tamoxifen resistance. To further increase confidence in our findings and rule out a cell line-specific effect, we measured the levels of SNHG6 in another ER-positive breast cancer cell line T47D. A comparison of levels in parental T47D vs. the tamoxifen resistant derivatives T47DTR revealed a >5-folds increase in SNHG6 levels in the resistant cells (**Figure 1B**). The increase in SNHG6 levels in both of the cell line pairs was found to be highly significant (p<0.01). As described in the Methods, resistant MCF7TR and T47DTR were generated by prolonged exposure of respective native cells to tamoxifen in cell culture set-up. To check whether such prolonged exposure of native cells to tamoxifen had indeed resulted in generation of tamoxifen-resistant derivatives, we checked the tamoxifen sensitivity of the paired cells. When exposed to increasing concentrations of tamoxifen for 48 hours, followed by MTT assay, we observed a remarkable increase (p<0.01) in the resistance against tamoxifen in ‘resistant’ cells, both MCF7TR (**Figure 1C**) and T47DTR (**Figure 1D**), against tamoxifen. The IC-50 for MCF7TR cells was >10-folds while the IC-50 for T47DTR cells was >8-folds, relative to the respective native cells (**Table 1**). In continuation of our observation that SNHG6 levels were elevated in resistant cells, we next silenced SNHG6 using LNA-modified siRNAs against lncRNA SNHG6. We first tested four different siRNA preparations (Results not shown) and chose the siRNA that demonstrated more than 80% downregulation of SNHG6. The testing was initially done in MCF7TR cells and the chosen siRNA was tested for its efficacy in T47DTR cells as well wherein, the siRNA again demonstrated more than 80% downregulation of SNHG6. siRNA against SNHG6 significantly sensitized MCF7TR cells against tamoxifen, as observed by significantly reduced (p<0.01) cell proliferation in the presence of tamoxifen (**Figure 1C**). Very similar results were observed in T47D cells, as well, with silencing of SNHG6 resulting in significantly reduced (p<0.01) proliferation when cells were treated with increasing concentrations of tamoxifen (**Figure 1D**).

**Table 1:** IC-50 values.

**Cell line**	**IC-50**	**Cell line**	**IC-50**
MCF-7	4.6 ± 0.1 μM	T47D	3.5 ± 0.2 μM
MCF7TR	> 50 μM	T47DTR	28.7 ± 0.8 μM
MCF7TR-siSNHG6	19.1 ± 0.6 μM	T47DTR-siSNHG6	10.0 ± 0.3 μM

The values were calculated based on the experiments reported in **Figures 1 C, D**.

### SNHG6 affects EMT, apoptosis, invasion and clonogenicity

**Figure 2:**
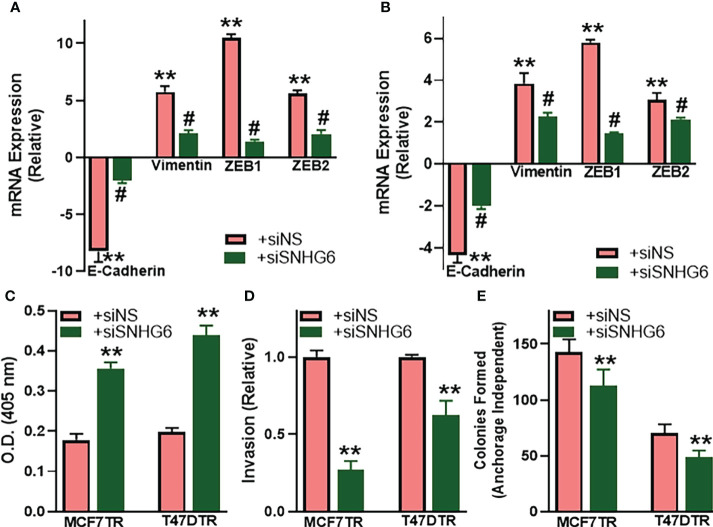
SNHG6 affects EMT, apoptosis, invasion and clonogenicity. **(A)** MCF7TR and **(B)** T47DTR cells were transfected with control siRNA (siNS) or siRNA against SNHG6 and then the levels of EMT markers, E-cadherin, vimentin, ZEB1 and ZEB2 were evaluated by qRT-PCR. The levels of these markers in respective parental cells were assigned a value of 1 and the relative levels in siNS/siSNHG6 transfected tamoxifen-resistant cells are plotted. **(C)** Apoptosis was assessed in tamoxifen-resistant cells by Histone/DNA ELISA method, as described in the Methods, by measuring the O.D. at 405 nm. **(D)** Invasive potential of tamoxifen-resistant cells was evaluated by quantitating the fluorescence of cells that invaded through the matrigel-coated membranes. Fluorescence of siNS cells assigned a value of 1 and the relative fluorescence of siSNHG6 transfected tamoxifen-resistant cells is plotted. **(E)** Clonogenicity was measured by counting the colonies formed by cells in an anchorage-independent manner, as described in the Methods. **p<0.01, compared to respective controls, #p<0.01, compared to siNS.

As a mechanism of tamoxifen resistance possibly induced by SNHG6, we first evaluated the process of epithelial-to-mesenchymal transition (EMT) because of the published reports demonstrating a profound modulation of EMT by SNHG6 (18, 19) and the role of EMT in tamoxifen resistance (20, 21). We evaluated the expression of EMT markers, E-cadherin, vimentin, ZEB1 and ZEB2 in paired cell lines. While E-cadherin is a marker of epithelial phenotype, the rest three are markers of mesenchymal phenotype. We observed that E-cadherin was markedly downregulated (p<0.01) whereas vimentin, ZEB1 and ZEB2 were markedly upregulated (p<0.01) in MCF7TR cells, relative to the parental MCF-7 cells, indicating the induction of EMT (**Figure 2A**). Similar induction of EMT was apparent in T47DTR cells as well, relative to parental T47D cells (**Figure 2B**), as evidenced by significantly downregulated (p<0.01) E-cadherin and significantly upregulated (p<0.01) vimentin, ZEB1 and ZEB2. We further studied the effect of silencing SNHG6 in these resistant cells to check if the silencing of oncogenic SNHG6 can reverse EMT. As shown in **Figures 2A, B**, we observed that silencing of SNHG6 significantly (p<0.01) attenuated the tamoxifen resistance-associated changes in EMT markers in both of the cell lines. The reduced levels of E-cadherin were attenuated by silencing of SNHG6 while the elevated levels of vimentin, ZEB1 and ZEB2 in tamoxifen-resistant cells were significantly reduced by the silencing of SNHG6. Further, silencing of SNHG6 induced apoptosis in both of the resistant cell lines (**Figure 2C**) in addition to reducing the invasion potential (**Figure 2D**) and the colony forming ability (clonogenicity) (**Figure 2E**) of both cell lines. These results suggest that SNHG6 silencing sensitizes tamoxifen resistant cells to tamoxifen through reversal of EMT, induction of apoptosis and associates with reduced aggressiveness and significantly reduced invasion potential and colony forming ability.

### Identification of miR-101 as the miRNA sponged by SNHG6

**Figure 3:**
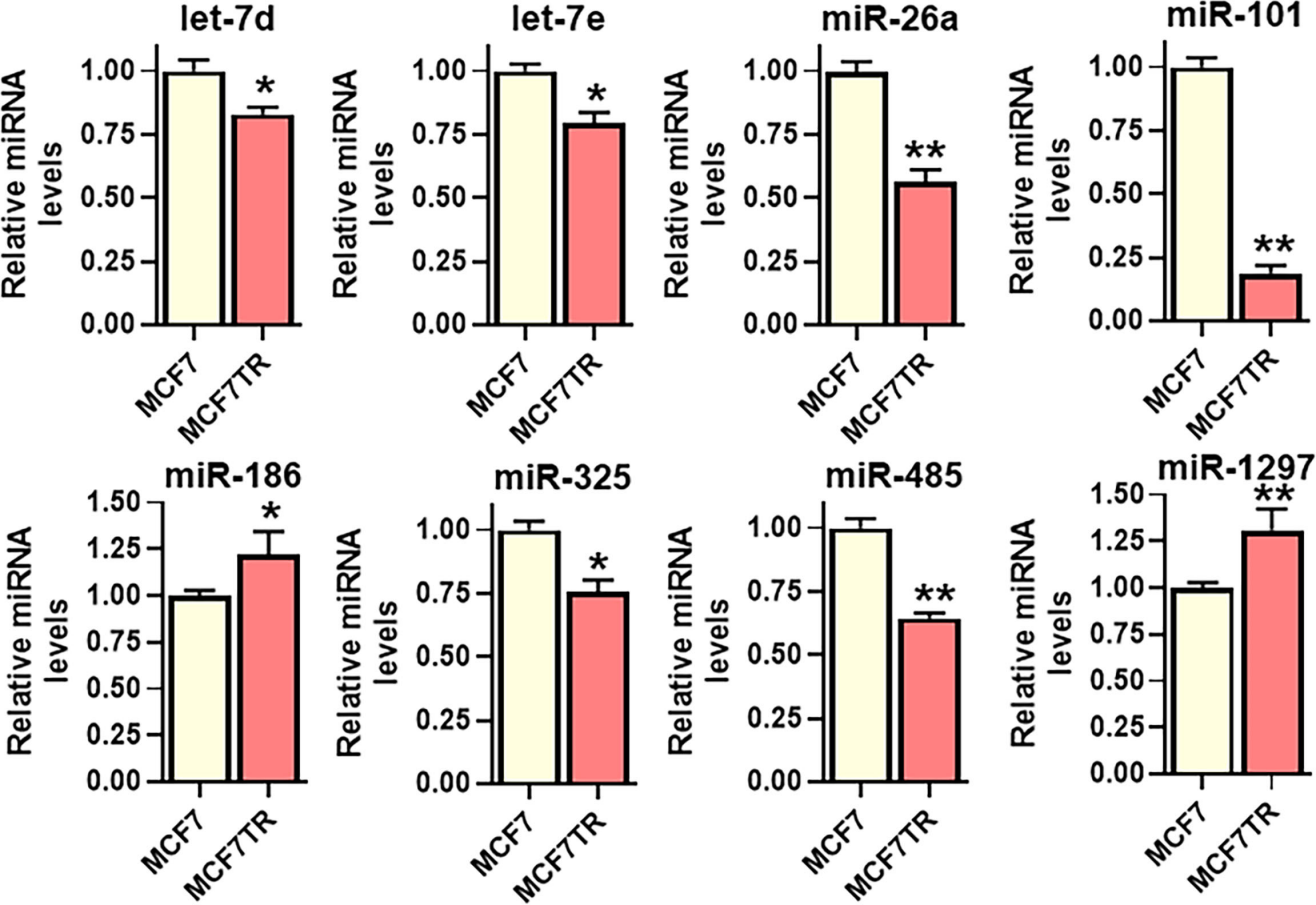
Identification of SNHG6 target miRNAs. Several miRNAs, as identified, were quantitated in MCF-7 and MCF7TR cells, using qRT-PCR. The levels of miRNAs in parental MCF-7 cells were assigned a value of 1 and the relative levels in tamoxifen-resistant MCF7TR are plotted. *p<0.05 and **p<0.01, compared to respective controls.

lncRNAs function through sponging of target miRNAs and, therefore, we next evaluated the miRNAs that are sponged by SNHG6 as evidenced through their negative regulation by SNHG6. For the screening of putative miRNAs, we searched the literature for published miRNAs that are sponged by SNHG6, and, additionally, employed bioinformatics-based approach to list the putative miRNAs that can be sponged. DIANA-LncBase v3 was used to list such miRNAs. A number of miRNAs were shortlisted and tested but only those that were significantly modulated in MCF7TR cells, relative to native MCF-7 cells, are reported in **Figure 3**. miR-101 (also referred to as miR-101-3p) was the most significantly (p<0.01) downregulated miRNA among all the tested miRNAs. Several other miRNAs were also found to be downregulated significantly (let-7d, let-7e and miR-325 with p<0.05 and miR-26a and miR-485 with p<0.01) whereas two miRNAs (miR-186 and miR-1297) were found to be upregulated in MCF7TR cells, relative to the MCF-7 cells. Based on these results, we chose miR-101 as the miRNA of interest for further mechanistic studies.

### miR-101 effects on tamoxifen resistance in MCF-7 cells

**Figure 4:**
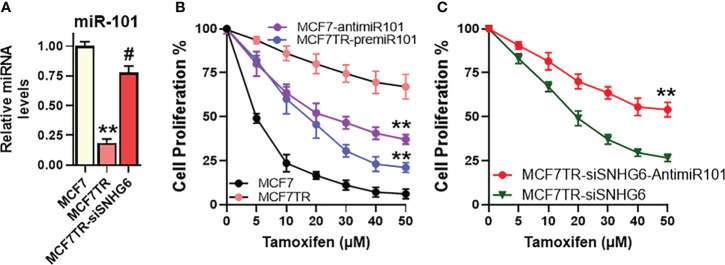
miR-101 affects tamoxifen resistance. **(A)** Levels of miR-101 were assessed in MCF-7, MCF7TR and MCF7TR cells silenced for SNHG6. Levels of miR-101 in parental MCF-7 cells were assigned a value of 1 and the relative levels in other cells are plotted. **(B)** Cell proliferation was measured by MTT assay in MCF-7/MCF7TR cells after transfections with pre/anti-miR-101 oligomers, as appropriate, followed by exposure to indicated concentrations of tamoxifen for 48 hours. **(C)** Tamoxifen resistant MCF7TR either just silenced for SNHG6 or additionally transfected with anti-miR-101 oligomers were treated with increasing concentrations of tamoxifen for 48 hours before the proliferation was assessed using MTT assay. **p<0.01, compared to respective controls, #p<0.01, compared to MCF7TR.

The effect of miR-101 on tamoxifen sensitivity was checked by manipulating the levels of miR-101 in MCF-7 as well as MCF7TR cells. First, we checked the levels of miR-101 in these two paired cells and found significantly reduced (p<0.01) miR-101 levels in MCF7TR cells, compared to the parental MCF-7 cells (**Figure 4A** **)**. We also checked for a regulation of miR-101 by SNHG6 in our experimental model and observed that silencing of SNHG6 significantly (p<0.01) attenuated the tamoxifen resistance-associated down-regulation of miR-101 in MCF7TR cells (**Figure 4A**). Since the levels of miR-101 were relatively higher in MCF-7 cells, we downregulated miR-101 levels in these cells, through the use of specific anti-miR-101 oligomers and subjected the cells to tamoxifen treatment for 48 hours. As shown in **Figure 4B**, such downregulation of miR-101 significantly increased the resistance of MCF-7 cells, relative to the MCF-7 cells with non-specific control oligomers. As an experiment to further confirm the role of miR-101 in tamoxifen resistance, we upregulated miR-101 in MCF7TR cells through transfections with pre-miR-101 and observed significantly reduced tamoxifen resistance (**Figure 4B**). Next, we checked for the functional relevance of miR-101 upregulation in SNHG6-silenced MCF7TR cells through the evaluation of tamoxifen sensitivity and found that antagonizing such elevated levels of miR-101 in SNHG6-silenced MCF7TR cells, through anti-miR-101 oligomers, significantly increased the resistance of MCF7TR cells against tamoxifen (**Figure 4C**).

### miR-101 effects on SNHG6-mediated EMT and cancer stem cells

**Figure 5:**
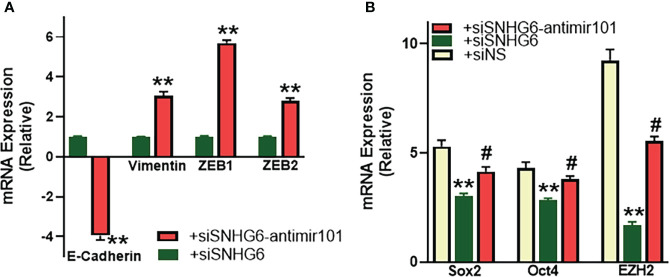
miR-101 affects EMT and cancer stem cell markers. **(A)** MCF7TR cells were silenced for SNHG6 and then the levels of EMT markers, E-cadherin, vimentin, ZEB1 and ZEB2 were evaluated by qRT-PCR in these cells as well as cells that were additionally transfected with anti-miR-101 oligomers. The levels of markers in SNHG6-silenced cells were assigned a value of 1 and the relative levels in siSNHG6+anti-miR-101-transfected cells are plotted. **(B)** Molecular markers of cancer stem cell phenotype, Sox2, Oct4 and EZH2 were quantitated, using qRT-PCR. The levels of miRNAs in parental MCF-7 cells were assigned a value of 1 and the relative levels in tamoxifen-resistant control MCF7TR (siNS) and those silenced for SNHG6 with and without additional transfections with anti-miR-101, are plotted. **p<0.01, compared to siNS, #p<0.01, compared to siSNHG6.

As reported above, SNHG6 had modulating effects on EMT induction, therefore, we next checked the mechanistic involvement, if any, of miR-101, the miRNA sponged by SNHG6, in the process. As shown in **Figure 5A**, relative to MCF7TR cells silenced for SNHG6, the cells with added anti-miR-101 oligomers had significantly reduced (p<0.01) epithelial marker E-cadherin levels along with significantly elevated (p<0.01) mesenchymal markers vimentin, ZEB1 and ZEB2. Also, in view of the intricate connection between drug resistance, EMT and the cancer stem cell phenotype (22, 23), we evaluated various markers of cancer stem cell phenotype in our experimental model system and found that all the tested markers, Sox2, Oct4 as well as EZH2 were significantly downregulated in SNHG6-silenced MCF7TR cells, relative to the control MCF7TR cells (**Figure 5B**). Transfection of anti-miR-101 oligomers in SNHG6-silenced MCF7TR cells resulted in significant (p<0.01) attenuation of SNHG6 silencing effects (**Figure 5B**) which further established the mechanistic role of miR-101 in SNHG6 mediated effects.

### miR-101 mediates SNHG6 effects in T47D cells as well

**Figure 6:**
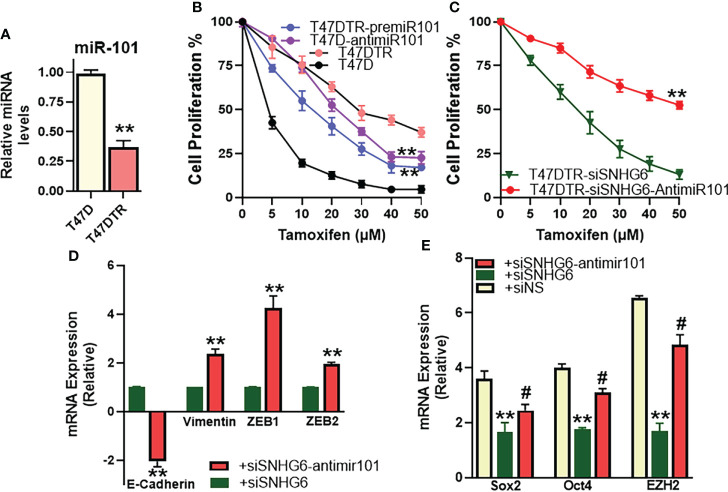
miR-101 affects validated in T47D cells. **(A)** Levels of miR-101 were assessed in T47DTR cells, relative to levels in parental T47D cells. Levels of miR-101 in parental cells were assigned a value of 1 and the relative levels in tamoxifen-resistant cells are plotted. **(B)** Cell proliferation was measured by MTT assay in T47D/T47DTR cells after transfections with pre/anti-miR-101 oligomers, as appropriate, followed by exposure to indicated concentrations of tamoxifen for 48 hours. **(C)** Tamoxifen resistant T47DTR either just silenced for SNHG6 or additionally transfected with anti-miR-101 oligomers were treated with increasing concentrations of tamoxifen for 48 hours before the proliferation was assessed using MTT assay. **(D)** T47DTR cells were silenced for SNHG6 and then the levels of EMT markers, E-cadherin, vimentin, ZEB1 and ZEB2 were evaluated by qRT-PCR in these cells as well as cells that were additionally transfected with anti-miR-101 oligomers. The levels of markers in SNHG6-silenced cells were assigned a value of 1 and the relative levels in siSNHG6+anti-miR-101-transfected cells are plotted. **(E)** Molecular markers of cancer stem cell phenotype, Sox2, Oct4 and EZH2 were quantitated, using qRT-PCR. The levels of miRNAs in parental T47D cells were assigned a value of 1 and the relative levels in tamoxifen-resistant control T47DTR (siNS) and those silenced for SNHG6 with and without additional transfections with anti-miR-101, are plotted. **p<0.01, compared to respective controls, #p<0.01, compared to siSNHG6.

We identified miR-101 as the miRNA sponged by SNHG6 in experiments that were carried out using MCF7 and MCF7TR cells, and further established the mechanistic role of miR-101 in SNHG6 effects in those cells. To further validate these findings, we performed similar experiments in T47D cells as well. We started with an evaluation of the relative levels of miR-101 in parental and tamoxifen resistant T47D cells, and observed significantly reduced (p<0.01) miR-101 levels in T47DTR cells (**Figure 6A**) thus confirming the earlier results from paired MCF7 cells. miR-101 manipulations had a profound effect on tamoxifen sensitivity as antagonizing miR-101 in parental T47D cells resulted in significantly (p<0.01) induced tamoxifen resistance while overexpression of miR-101, through pre-miR-101 oligomers, resulted in significant (p<0.01) sensitization of T47DTR cells to tamoxifen (**Figure 6B**). In context of the mechanistic involvement of miR-101 in SNHG6 induced tamoxifen resistance, anti-miR-101 oligomers significantly (p<0.01) increased the sensitivity to tamoxifen of T47DTR cells silenced for SNHG6 (**Figure 6C**) which involved modulation of SNHG6-mediated EMT because, relative to T47DTR cells with silenced SNHG6, the ones that additionally were transfected with anti-miR-101, showed induction of EMT, as evidenced by downregulated E-cadherin and upregulated vimentin, ZEB1 and ZEB2 (**Figure 6D**). Further, SNHG6 silencing reduced cancer stem cell markers Sox2, Oct4 and EZH2 in T47DTR cells, relative to parental T47D cells, and anti-miR-101 oligomers significantly (p<0.01) attenuated this effect (**Figure 6E**).

## Discussion

Acquired resistance against tamoxifen is a major clinical challenge that severely impacts the clinical management of ER-positive breast cancer patients. A number of lncRNAs are now known to play a role in resistance against cancer therapies (24, 25), including resistance against tamoxifen (7, 10). In the present study, we focused on lncRNA SNHG6 for its potential role in acquired tamoxifen resistance in ER-positive breast cancer cells. Our hypothesis to focus on SNHG6 for this study was based on the published literature supporting a role of SNHG6 in cancer drug resistance, coupled with the fact that its role specifically in tamoxifen resistance has never been elucidated. As per the published literature, SNHG6 can impact cisplatin resistance in gastric cancer (15, 26), 5-FU resistance in colorectal cancer (14), paclitaxel resistance in prostate cancer (16) as well as radio-resistance in cervical cancer (13). We now provide first evidence for a role of lncRNA SNHG6 in drug resistance of breast cancer, in general, and tamoxifen resistance, in particular.

We report elevated levels of SNHG6 in tamoxifen-resistant cells, which is in agreement with the published literature about the oncogenic role of SNHG6 in human cancers (12, 27, 28). Since SNHG6 is itself elevated in tamoxifen resistant cells, the miRNAs that it sponges are expected to be tumor-suppressive. In agreement with this, not only the miR-101 that we shortlisted for detailed mechanistic evaluation based on the observation, was the most down-regulated miRNA in tamoxifen resistant cells, but several other putative miRNAs, such as let-7d, let-7e, miR-26a, miR-325 and miR-485 were also found to be significantly downregulated. Incidentally, all of these miRNAs that were down-regulated in our experimental model, are known to be tumor suppressor miRNAs (29–33). Of note, we observed two individual miRNAs, miR-186 and miR-1297 to be upregulated which was a little confusing, given the earlier reports suggesting sponging of these miRNAs by SNHG6. However, both of these miRNAs seem to have a dual effect (34, 35), both oncogenic as well as tumor suppressive, and our results support their reported oncogenic function. It would also be of interest to readers to know that we combined the information from published literature as well as bioinformatics analysis to shortlist the potential miRNAs that can be sponged by SNHG6. miR-26a (14), miR-186 (16), miR-325 (15), miR-485 (13) and miR-1297 (26) represent miRNAs that were shortlisted based on published reports while let-7d/e and miR-101 represent miRNAs that were identified through DIANA-LncBase v3 platform.

One of the primary mechanism through which SNHG6 mediates its oncogenic effects is the induction of EMT. Accordingly, SNHG6 has been shown to induce EMT leading to increased proliferation/migration/invasion of gastric cancer cells (36), colorectal cancer cells (37, 38), pituitary adenoma (18), glioma (19) and even breast cancer cells (39). However, our work is the first to demonstrate induction of EMT by SNHG6 in breast cancer cells with functional implications in acquired resistance against tamoxifen. We show here that SNHG6 silencing reversed EMT leading to acquisition of a tamoxifen sensitive phenotype. This is a clear proof supporting our hypothesis that SNHG6 plays a role in tamoxifen resistance. Further, we demonstrate a negative correlation between SNHG6 and miR-101 levels. In the SNHG6-silenced cells, miR-101 levels are higher, which makes sense given the tumor suppressive nature of miR-101. Furthermore, antagonizing miR-101 in SNHG6 silenced cells once again induced EMT, as evidenced by downregulated epithelial marker E-cadherin and the upregulated mesenchymal markers, vimentin, ZEB1 and ZEB2, thus supporting EMT induction as well as significantly increased tamoxifen resistance.

While our present report is the first one to demonstrate sponging of miR-101 by SNHG6 in breast cancer, particularly in tamoxifen-resistant breast cancer, there is ample evidence from other cancer as well as non-cancer models to confirm such miR-101 sponging by SNHG6. In one of the early report on the subject, SNHG6 was reported to sponge miR-101 in hepatocellular carcinoma cells (40). This work also reported an effect of SNHG6 on mesenchymal marker ZEB1, similar to one shown in our present study. Thereafter, SNHG6 was shown to sponge miR-101 in gastric (36), glioma (41), colorectal (42), non-small cell lung (43), cholangiocarcinoma (44), melanoma (45) and esophageal (46) cancer cells. In addition, SNHG6 has been reported to sponge miR-101 in rat degenerate nucleus pulposus cells (47) and such sponging of miR-101 by SNHG6 has been suggested to contribute to ventricular septal defect formation (48). Thus, specific targeting of miR-101 by SNHG6 is functionally relevant not only in various cancers but in several other physiological phenomena as well. Our novel information on the modulation of tamoxifen resistance by this SNHG6-miR-101 axis adds new knowledge, and should further generate interest in the evaluation of miR-101 targeting by SNHG6 in the context of cancer drug resistance.

In our present work, we also report that miR-101 itself affected tamoxifen resistance. Just the manipulation of miR-101 levels in parental as well as tamoxifen-resistant cells, through the use of pre- or anti-miR-101 oligomers, as appropriate, resulted in differential sensitization of cells to tamoxifen exposure. More importantly, we determined miR-101 to be mechanistically involved in SNHG6 effects because miR-101 was found to attenuate the SNHG6 effects on tamoxifen sensitization/resistance in both MCF-7/MCF7TR and T47D/T47DTR paired cell lines. We thus provided novel evidence to support a role of miR-101 in tamoxifen resistance of ER-positive breast cancer. An earlier report has documented an estrogen-independent growth stimulation by miR-101 in MCF-7 cells (49). There, however, has been no attempt yet to connect miR-101 with tamoxifen resistance of breast cancers, which underlines another important revelation from our work.

In our experiments, we found ZEB1 to be the most affected EMT biomarker. It was found to be the most differentially regulated molecular marker, among all the markers of EMT tested, in the tamoxifen resistant cells, when compared to the parental cells. ZEB1, also happened to be to most affected EMT marker when SNHG6 was silenced in tamoxifen-resistant cells and finally, ZEB1 was again the most affected EMT marker when anti-miR-101 oligos were added to SNHG6-silenced cells. In a similar observation, EZH2 was found to be the most affected cancer stem cell marker as it was found to be the most up-regulated stem cell marker in tamoxifen-resistant cells, most affected marker in SNHG6-silenced cells and then the best marker rescued by miR-101 manipulation in SNHG6-silenced cells. Interestingly, miRDB prediction lists ZEB1 and EZH2 as the predicted targets of miR-101 which is also supported by published evidence of ZEB1 (40) and EZH2 (46) targeting by miR-101. Taken together, our study provides evidence to support a role of lncRNA SNHG6-miR101 axis in tamoxifen resistance of ER-positive breast cancers which involves modulation of EMT and cancer stem cells phenotype through targeting of ZEB1/EZH2. Further clinical studies will be needed to exploit this novel information for future benefit of breast cancer patients, particularly those with acquired tamoxifen resistance.

## Data availability statement

The original contributions presented in the study are included in the article. Further inquiries can be directed to the corresponding author.

## Author contributions

Conceptualization, validation, resources, writing—review and editing, supervision, and project administration, AA. Methodology, validation, investigation, data curation, and writing—original draft preparation, MK and AA. Formal analysis and funding acquisition, MK. Both authors have read and agreed to the published version of the manuscript.

## Funding

The authors extend their appreciation to the Deputyship for Research & Innovation, Ministry of Education in Saudi Arabia for funding this research work through the project number IFPIP: 1051-130-1442 and King Abdulaziz University, DSR, Jeddah, Saudi Arabia.

## Conflict of interest

The authors declare that the research was conducted in the absence of any commercial or financial relationships that could be construed as a potential conflict of interest.

## Publisher’s note

All claims expressed in this article are solely those of the authors and do not necessarily represent those of their affiliated organizations, or those of the publisher, the editors and the reviewers. Any product that may be evaluated in this article, or claim that may be made by its manufacturer, is not guaranteed or endorsed by the publisher.
